# 2-methoxyestradiol sensitizes tamoxifen-resistant MCF-7 breast cancer cells via downregulating HIF-1α

**DOI:** 10.1007/s12032-024-02471-w

**Published:** 2024-08-21

**Authors:** Yasmin M. Attia, Hamada Ahmed Mokhlis, Ahmed Ismail, Ahmed S. Doghish, Mohamed H. Sobhy, Sherif S. Hassanein, Walaa A. El-Dakroury, Amr D. Mariee, Salama A. Salama, Marwa Sharaky

**Affiliations:** 1 Pharmacology Unit, Cancer Biology Department, National Cancer Institute, Cairo University, Kasr El Aini Street, From El Khalig, Cairo, 11796 Egypt; 2 Faculty of Pharmacy (Boys), Department of Pharmacology and Toxicology, Al-Azhar University, Nasr City, Cairo, 11231 Egypt; 3 Faculty of Pharmacy, Department of Pharmacy Practice, Sinai University, Al Qantarah Sharq, 41636 Ismailia Governorate Egypt; 4 Faculty of Pharmacy (Boys), Department of Biochemistry and Molecular Biology, Al-Azhar University, Nasr City, Cairo, 11231 Egypt; 5 Faculty of Pharmacy, Department of Biochemistry, Badr University in Cairo (BUC), Badr City, Cairo, 11829 Egypt; 6 Faculty of Pharmacy, Al-Azhar University, Nasr City, Cairo, 11231 Egypt; 7 Nanomedicine Research Labs, Center for Materials Science, Zewail City of Science and Technology, 6th of October City, Giza, Egypt; 8 Faculty of Pharmacy, Department of Pharmaceutics and Industrial Pharmacy, Badr University in Cairo (BUC), Badr City, Cairo, 11829 Egypt

**Keywords:** Breast cancer, Tamoxifen-resistance, LCC2, 2-Methoxyestradiol, HIF-1α

## Abstract

The clinical studies for breast cancer (BC) are now assessing the efficacy of 2-Methoxyestradiol (2-ME), a naturally occurring derivative of estradiol. Our study aimed to explore the potential of combining the 2-ME and tamoxifen (TAM) on sensitization of TAM-resistant cells using LCC2 the TAM-resistant cells as a model and comparing the results to the sensitive cells MCF-7. Sulphorhodamine-B (SRB) assay is used to examine the 2-ME chemo-sensitizing impact on the cytotoxicity of TAM on LCC2 cells. Colorimetric assay kits were used to assess the level of the apoptosis-related markers caspases 3, Bcl2, and Bax in cell lysate. Hypoxia-inducible factor 1 alpha (HIF-1α) expression was measured using western blotting. Total cholesterol and triglyceride (TG) levels were examined colorimetrically, using the BIOLABO kit. The use of 2-ME enhanced the cytotoxic effects of TAM and effectively reversed TAM resistance. This was achieved by inhibiting the expression of HIF-1α, while concurrently increasing the levels of apoptotic marker caspase-3, as well as the pro-apoptotic protein Bax. Additionally, there was a reduction in the levels of Bcl2, an anti-apoptotic protein. Furthermore, a reduction in TG and cholesterol levels was noted. Our findings show that HIF-1α plays an important role in TAM resistance and that suppression of HIF-1α by 2-ME-mediated sensitization of BC-resistant cells to TAM. Therefore, the concurrent administration of TAM/2-ME might potentially serve as a viable therapeutic approach to address TAM resistance and enhance the overall therapy efficacy for patients with BC.

**Supplementary Information:**

The online version contains supplementary material available at 10.1007/s12032-024-02471-w.

## Introduction

Breast cancer is the second cause of cancer death among female [1]. Breast cancer is classified into four forms based on different gene expressions that convey hormone receptor status: estrogen receptor-positive (ER^+^) BC, which includes luminal types A and B, triple-negative/basal-like (TNBC), and HER-2 dependent cellular subtypes. The ER^+^ cellular subtypes contribute to nearly seventy percent of all BC cases [2]. Endocrine treatments are the cornerstone in the therapy of hormone-dependent BC, and they act either by modifying or interrupting the synthesis of estrogen and the existence or action of ERs [3]. The most used hormone therapy for BC is TAM.

Tamoxifen (TAM) continues to be a vital pharmacological agent in the treatment of several cancer types, notably BC. TAM is the initial selective estrogen receptor modulator (SERM) available for clinical use. TAM acts as antiestrogenic via estrogen receptor alpha (ERα) [4]. However, the development of resistance significantly reduces its efficacy and application [5]. There are plenty of complex biochemical mechanisms underlying TAM resistance in cancer cells, as reported ERα levels in resistant cells are lower than in normal BC cells, and the hypoxic tumor microenvironment has long been linked to resistance [6]. TAM is one of the most well-known regulators of hypoxia-inducible factor 1 alpha (HIF-1α) as it is reported to reduce the levels of HIF-1α and estrogen receptor alpha (ER-α) in many cancer cells including BC and pancreatic cancer [7]. The HIF-1α protein degrades quickly under normal circumstances, but under hypoxic conditions, it stabilizes and is upregulated. Furthermore, the cross-talk between ERα and HIF-1α is well known for example, it was shown that ER-α agonists such as 17-β estradiol (E2) significantly increases the expression of HIF-1α under hypoxic conditions while fulvestrant an ER-α blocker diminish the expression of HIF-1α under the same condition [7].

The hypoxic tumor microenvironment that stabilizes the HIF-1α is responsible for the great loss of ER-α protein in BC cell membranes by the manner of proteolysis rather than inhibiting its transcription [8]. Additionally, HIF-1α is associated with poor prognosis in TAM-resistant BC cell lines where HIF-1α levels were increased compared to naïve tumors [9]. It is believed that the proteins in the Bcl-2 family, such as anti-apoptotic Bcl-2 and pro-apoptotic BAX are dysregulated in BC and contribute to tamoxifen resistance [10]. In addition, TAM is known to induce apoptosis in BC cells, and pretreatment with caspase 3 or general caspase inhibitors prevents TAM-induced apoptosis [11].

2-Methoxyestradiol (2-ME) is a naturally occurring estrogenic metabolite that is well-tolerated and has diverse pharmacological activities. It has the potential to be a chemosensitizer because it addresses the chemoresistance pathway on several levels. It blocks hypoxia-induced and constitutive HIF-1α, blocking HIF-1α nuclear accumulation and HIF-transcriptional activity [12]. Despite 2-ME being an estrogenic metabolite, however, it binds poorly to ERα and induces apoptosis apart from the expression of ERα in the cancer cell membrane which makes it a great candidate when it comes to ER-negative and resistant cancer cells [13]. Moreover, it was reported that 2-ME selectively increases the susceptibility of radioresistant MCF-7/FIR cells to gamma radiation by focusing on many signaling pathways that contribute to the formation of radio-resistance [14]. Furthermore, 2-ME chemosensitizer doxorubicin-resistant MCF-7 by lowering BCl-2, cyclin D1, stimulating caspase 3 and causing a cell cycle halt in the G and S stages [15]. Since it has been indicated that an increased ratio of pro-apoptotic BAX protein to anti-apoptotic Bcl-2 protein expression can be associated with apoptosis, it’s reported that 2-ME increases BAX/Bcl-2 ratio in some cancer cells [16]. Remarkably, recent research has shown that 2-ME stimulates alternative modes of cell death, such as interferon-mediated autophagy, in addition to its apoptotic effect, which may be compromised in chemo-resistant cells. Above all, 2-ME has highly essential properties, for instance, it has the ability to distinguish between normal and cancer cells [17]. Yet, tamoxifen resistance is still a clinical concern for hormone receptor-positive BC. So, the current study aimed to explore the potential of combining the 2-ME and TAM on sensitization of TAM-resistant cells using LCC2 the TAM-resistant cells as a model, and comparing the results to the sensitive cells MCF-7.

## Materials and methods

### Chemicals and reagents

Tamoxifen (TAM) was acquired from Amria Pharmaceuticals Company (Alexandria, Egypt). DMSO was used to dissolve tamoxifen, and 2-ME was purchased from Sigma Aldrich (St. Louis, MO, USA) and dissolved in salt water. Both TAM and 2-ME were stored in a stock solution at −20 °C. Both TAM and 2-ME were serially diluted in RPMI1640 immediately before use, with final concentrations ranging from 2.5 to 100 M and 1.25 to 10 M, respectively.

Sigma Aldrich Chemical (St. Louis, MO, USA) was the exclusive supplier of all components, solvents, and reagents. (Milan, Italy-based) Sigma was kind enough to provide the RIPA lysis buffer. Thermofisher (Waltham, Massachusetts, United States) was where we got our fetal bovine serum, PBS, penicillin/streptomycin solution, RPMI-1640, and trypsin–EDTA.

### Cell lines

Human BC cell line MCF-7 was obtained from the American Type Culture Collection (ATCC; USA) and cultivated at the National Cancer Institute (NCI; Egypt) in RPMI1640 medium supplemented with 10% fetal bovine serum (FBS) and 1% penicillin–streptomycin. Robert Clarke (Georgetown University Medical Center, Washington, DC, USA) generously supplied the tamoxifen-resistant MCF-7, LCC2. [18]. Then the resistance was confirmed by cytotoxicity [19]. Every cell line was maintained in a constant humid 37 °C incubator with 5% CO2.

### Cytotoxicity assay

The anticancer activities of TAM and 2-ME on BC cells were evaluated using the Sulphorhodamine-B (SRB) assay [20]. Cells were sown in 96-well microtiter plates at a density of 3 × 10^3^ cells per well. Prior to drug incubation, a 24-h period was allocated for cell attachment. Subsequently, the cells were subjected to different doses of TAM ranging from 2.5 to 15 µM for MCF-7 cells and 10 to 100 µM for LCC2 cells, as well as 1.25 to 10 µM of 2-ME for both MCF-7 cells and their counterpart. This exposure lasted for a duration of 48 h. A control vehicle consisting of 0.1% v/v of DMSO was used. The cells were subjected to staining using a 0.4% solution of SRB dye, followed by fixation using a 20% concentration of trichloroacetic acid after the incubation period. The optical density (OD) of each well was measured spectrophotometrically at a wavelength of 570 nm using a TECAN sunriseTM ELISA reader from Germany.

The survival fraction may be calculated by dividing the OD of treated cells by the OD of control cells. The determination of the IC50 (concentration that inhibits cell growth by 50%) for each medication was performed using GraphPad Prism 8, via the use of sigmoidal dose–response curve-fitting models. Various concentrations of 2-ME were combined with the quarter and half of the IC50 value of TAM for the combination (ranging from 1.25 to 10 µM). Furthermore, the CompuSyn program was used to compute the combination index (CI) [21–23] to assess the extent of interaction between TAM and 2-ME on LCC2 and MCF-7 cells. Following the calculation of CI, the lowest concentrations given the smaller CI value were used for subsequent experiments. Therefore, this work conducted mechanistic studies by using a concentration of 2.5 μM TAM (half of the IC50) and/or 2.5 μM 2-ME on MCF-7 cells, as well as 35 μM TAM and/or 1.25 μM 2-ME on LCC2 cells, for a duration of 48 h.

### Assay of caspases 3, Bcl2, and Bax

In accordance with the guidelines provided by the manufacturer, colorimetric assay kits (Cloud-clone Corp, Houston, TX, USA) were used to evaluate the expression levels of apoptosis-related markers, namely Bcl2, caspases 3, and Bax, in cell lysate. The measurements were conducted at a wavelength of 450 nm using specific product numbers for each marker: SEA626Hu for Caspase 3, SEA778Hu for Bcl2, and SEB343Hu for Bax. Following a 24-h period, a total of 8*10^5^ cells were introduced into individual wells of 6-well plates. The cells were treated with TAM, 2-ME, and a combination of both compounds for a duration of 48 h. Following the incubation time, the cell pellets were lysed using protein lysis buffer, and subsequently, the apoptotic markers were evaluated. The identification of changes in Bcl2, caspases 3, and Bax was achieved by a comparison of the data with the untreated control level. The experiment was conducted in triplicate, with each trial being performed independently.

### Determination of protein concentration

The cells were subjected to trypsinization, followed by collection and subsequent storage at a temperature of −80 °C. This was done after exposing the cells to TAM and/or 2-ME for 48 h. Subsequently, cellular lysis was achieved by using a RIPA lysis buffer supplemented with protease inhibitors. The RIPA lysis solution consisted of 25 mM Tris HCL at pH 7.6, 150 mM NaCl, 1% triton X-100, 1% sodium deoxycholate, and 0.1% SDS. The protein contents in the cell lysate were measured using the Bradford assay kit obtained from Pierce (Rockford, IL, USA) [24].

### Western blot analysis

In summary, the RIPA buffer was used for cell lysis, followed by the transfer of the lysed cells to an Eppendorf tube and subsequent centrifugation at a speed of 13,000 revolutions per minute for a duration of 15 min. The proteins extracted were separated on a PVDF membrane using SDS-PAGE with a 12 percent acrylamide gel. The membranes were probed using the mouse monoclonal anti-HIF-1α primary antibodies (Santa Cruz Biotechnology, Inc., CA, USA) at a dilution of 1:500. Following an overnight incubation period, the membranes were subjected to a washing step and then incubated at room temperature for one hour. During this incubation, an alkaline phosphatase-conjugated goat anti-mouse secondary antibody (Novus Biologicals, LLC, Littleton, CO, USA) was used at a dilution of 1:5000. The commercially available kit was used to detect the antibody that is bound to the membrane. The program Win Image Studio Lite 5.2.5 was used to quantify band intensities. β-actin (Santa Cruz Biotechnology, Inc., CA, USA) was employed as a loading control at a dilution of 1:500.

### Determination of triglycerides (TG) and cholesterol

In the present study, LCC2 or MCF-7 cells were cultured in 6 well plates with a density of 8 × 10^5^ cells per well. After administering TAM and/or 2-ME therapy, the levels of total cholesterol and TG were analyzed using the BIOLABO kit (Les Hautes Rives, France) by a colorimetric method, following the directions provided by the manufacturer. The absorbance at a wavelength of 340 nm was measured using a spectrophotometer manufactured by Milton Roy Co., TX, USA. The amount of cholesterol and triglycerides was determined by performing calculations using the corresponding protein content.

### Statistical analyses

Three replicates of each experiment were performed independently. The data were presented as means ± SD and were subjected to analysis using GraphPad Prism 8. Post hoc tests were used in conjunction with ANOVA to assess the statistical disparities among the tested parameters. A significance level of *P* < 0.05 was considered to indicate statistical significance. The CompuSyn program was used to compute the combination index (CI) for quantifying the extent of interaction between 2-ME and TAM. The phenomenon of synergism is represented by CI value that is less than 1, while an additive effect is shown by a CI value equal to 1, and antagonism is characterized by a CI value greater than 1.

## Results

### TAM and 2ME reduce the viability of MCF-7 and LCC2 cells in a dose-dependent way

**Fig. 1:**
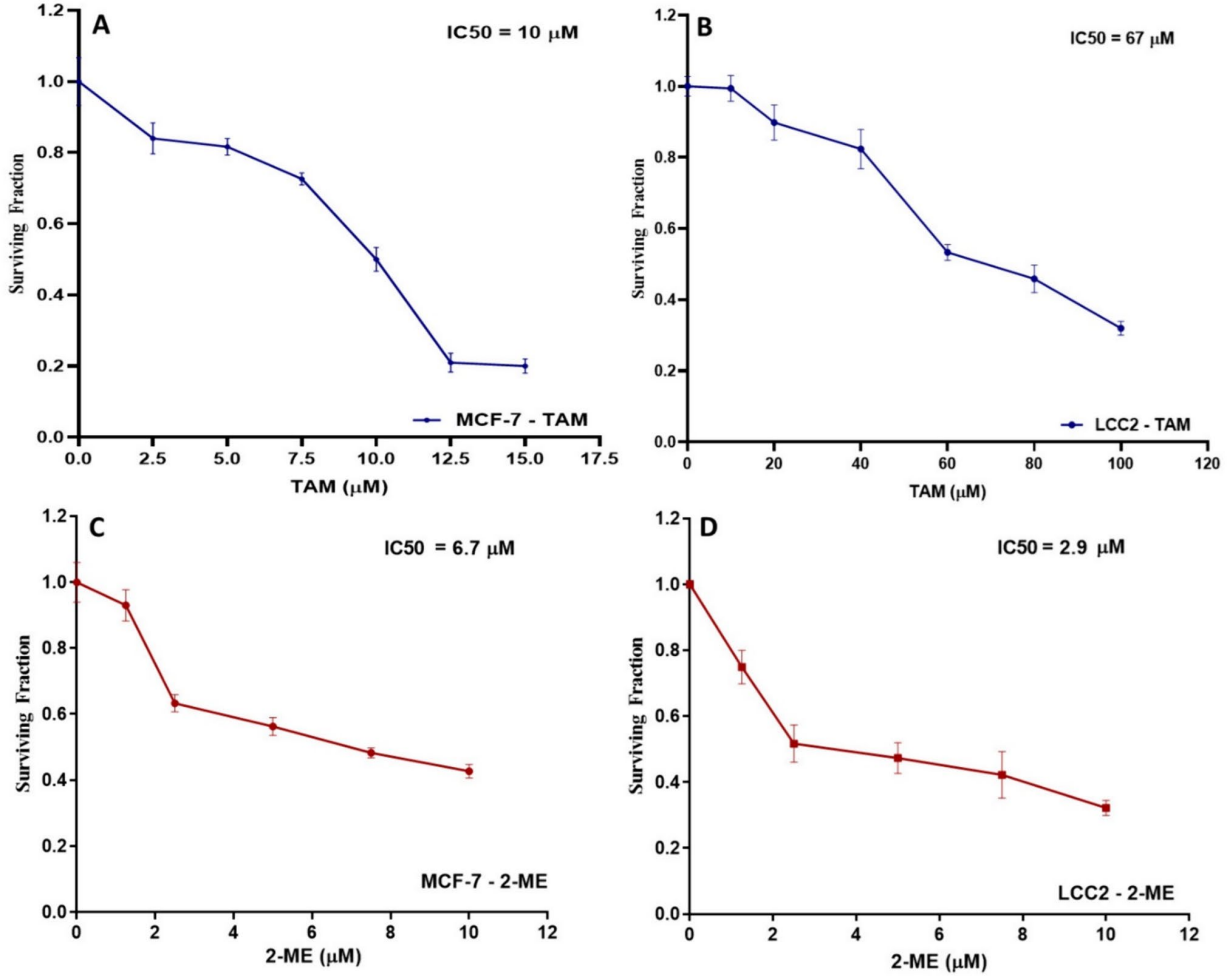
TAM and 2-ME suppress MCF-7 and LCC2 cell viability in a concentration-dependent manner. TAM reduces the viability of MCF-7 **A** and LCC2 **B** cells in a dose-dependent way. After a period of 48 h of being subjected to various TAM concentrations (2.5–15 μM for MCF-7 cells and 6.25–100 μM for LCC2 cells), the viability of the MCF-7 and LCC2 BC cell lines was assessed using the SRB test technique. The results, which were presented in relation to the control cells, were summarized as means ± SD obtained from three separate experiments, each of which was done in triplicate. The TAM IC50 was determined in both cells. Similarly, 2-ME decreases MCF-7 **C** and LCC2 **D** cell viability in a dose-dependent way. After 48 h of exposure to different 2-ME concentrations (0.625–10 μM for LCC2 and MCF-7 cells), the vitality of both cells was assessed using the SRB test technique. The results were presented as the mean ± SD derived from 3 consecutive experiments, each carried out in triplicate, and were given relative to the control cells. The 2-ME IC50 was detected in both cells

The SRB test method was used to examine the cytotoxicity of TAM in MCF-7 and LCC2 cells, identify the relative vitality of the cells, and calculate the IC50 of TAM in both cell lines. TAM (2.5–15 μM for MCF-7 cells and 10–100 μM for LCC2 cells) was applied to the cells for 48 h. MCF-7 and LCC2cell viability were lowered in a concentration-dependent manner. TAM IC50 values on MCF-7 and LCC2 cells were 10 μM and 67 μM, respectively (Fig. 1A, B). In the same manner, various concentrations of 2-ME (1.25–10 μM) were applied to MCF-7 and LCC2 cells over the course of 48 h. MCF-7 and LCC2 viability was decreased by 2-ME in a dose-dependent way. On MCF-7 and LCC2 cells, the IC50 for 2-ME was 6.7 μM and 2.9 μM, respectively (Fig. 1C, D).

### 2-ME improves TAM cytotoxic activity in MCF-7 cells

**Fig. 2:**
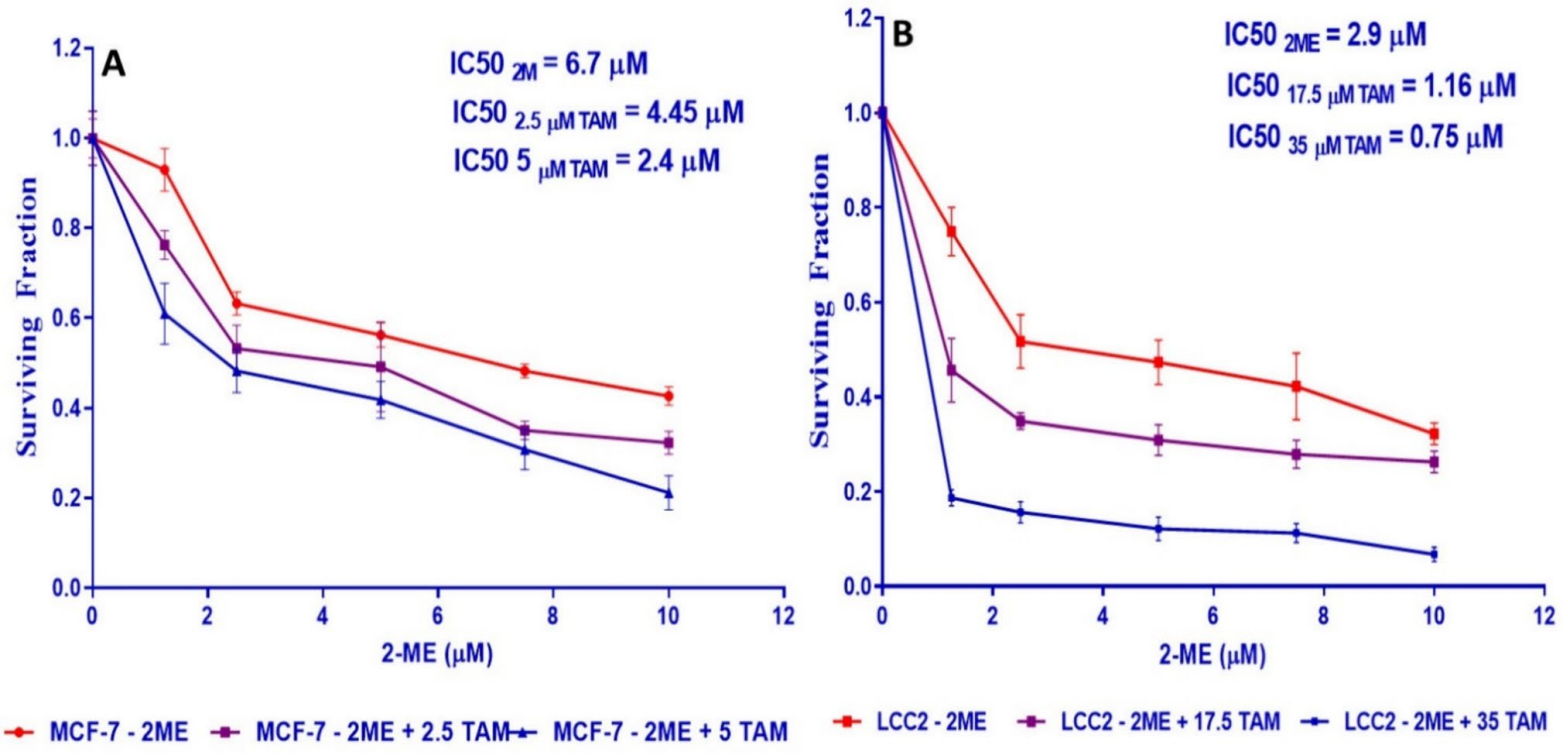
2-ME improves TAM’s cytotoxic impacts on MCF-7 and LCC2 cells. Effect of 2ME alone and in combination with TAM on dose–response in MCF-7 cells **A**. Exemplary SRB test demonstrating the interaction between two doses of TAM (2.5 μM and 5 μM) and various amounts of 2-ME in MCF-7 cells. Each line depicts the viability of cells following treatment with 2ME alone (●), with 2.5 μM (■), or with 5 μM (▲) of TAM. The concentration of 2ME is given on the -axis. Cell viability was assessed by contrasting it to the survival of cells in untreated (negative control) cultures, which was standardized to 1. Results are shown as mean ± SD from three separate trials. The 2ME IC50 alone and when coupled with TAM were calculated. **B**: In LCC2 cells, the dose–response correlation between TAM alone and coupled with 2ME. Typical SRB test demonstrating a relationship between two different concentrations of TAM citrate (17.5 μM and 35 μM) and different doses of 2ME in LCC2 cells. The 2ME amount is shown on the x-axis, and each line shows the survival of cells following treatment with 2ME alone (●), 17.5 μM (■), or 35 μM (▲) of TAM. The survival rate of cells in the negative control cultures, which was adjusted to 1, was used to compare cell viability. Results are shown as mean ± SD from three separate trials. The IC50 of 2ME singly and in combination with TAM were calculated

The IC50 of 2-ME in MCF-7 cells decreased and changed to 4.45 M and 2.4 μM, respectively, when combined with 2.5 μM and 5 μM TAM, as shown by the dose–response curve in Fig. 2A. Supplementary Table 1 shows the percentage of MCF-7 that is still viable following co-treatment with TAM at concentrations between 2.5 and 5 μM and different concentrations of 2-ME (0 μM, 0.625 μM, 1.25 μM, 2.5 μM, 5 μM, and 10 μM).

### 2-ME enhances TAM’s cytotoxic actions on LCC2 cells

Similarly, The curve of dose–response in Fig. 2B showed that when combined with 17.5 μM and 35 μM TAM, the IC50 concentrations of 2-ME in LCC2 cells decreased and shifted to 1.16 μM and 0.75 μM, respectively. Supplementary Table 1 shows how TAM (17.5 and 35 μM) and 2-ME (1.25, 2.5, 5, 7.5, and 10 μM) together affected the viability of LCC2 cells. TAM and 2-ME exhibited a notable cytotoxic impact on LCC2 cells, in contrast to how the two drugs were combined to treat MCF-7 cells.

### 2-ME enhances TAM efficiency in a synergistic manner

The CompuSyn tool was used to compute the CI to evaluate if the interaction between 2-ME and TAM on the survival of cells is cumulative, opposing, or synergistic. First, a synergistic effect was seen in MCF-7 cells where the CI between 2-ME and TAM was < 1, with a range of 0.62–0.98 (Table 1). Therefore, the lower concentration that revealed a reduced *CI* = 0.62, TAM (2.5 μM), and 2-ME (2.5 μM) were combined in the subsequent tests. Second, for LCC2 cells the CI of 2-ME and TAM was < 1, ranging from 0.23 to 0.87 in LCC2 cells, indicating a notable synergistic action (Table 1). As a result, the TAM (35 μM) and/or 2-ME (1.25 μM), revealed a reduced *CI* = 0.23 and were combined in the subsequent tests. These results showed that TAM cytotoxicity against both cells was enhanced synergistically when combined with 2-ME than solitary treatment.

**Table 1:** The synergistic effect of TAM and 2-ME on the viability of MCF-7 and LCC2 cells is indicated by the combination index (CI)

MCF-7			LCC2		
TAM (µM)	2-ME (µM)	CI	TAM (µM)	2-ME (µM)	CI
2.5	1.25	0.97	17.5	1.25	0.48
2.5	2.5	0.62	17.5	2.5	0.50
2.5	5	0.98	17.5	5	0.72
2.5	7.5	0.91	17.5	7.5	0.87
2.5	10	1.07	17.5	10	1.01
5	1.25	0.85	35	1.25	0.23
5	2.5	0.80	35	2.5	0.26
5	5	1.04	35	5	0.27
5	7.5	1.16	35	7.5	0.32
5	10	1.21	35	10	0.24

### Combined treatment of TAM/2-ME decreases HIF-1α protein expression levels in both MCF-7 and LCC2 cells

**Fig. 3:**
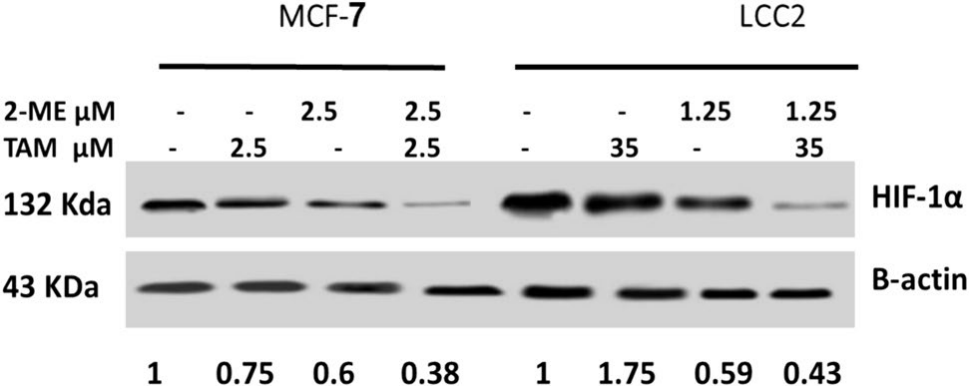
Combined treatment of TAM/2-ME decreases hypoxia-inducible factor alpha (HIF-1α) protein expression level in both MCF-7 and LCC2 cells. 2-ME/TAM co-treatment decreases HIF-1α expression in both MCF-7 and LCC2. Western blot analysis showed that the combination of 2-ME and TAM inhibited HIF-1α in both MCF-7 and LCC2 cells were treated with 2-ME (2.5 M, 1.25 µM, respectively) and TAM (2.5, 35 µM, respectively) for 48 h. Numbers under the bands represent the densitometric analysis of the western band compared to β-actin control

The impact of therapy with TAM (2.5, 35 µM) and/or 2-ME (2.5 µM, 1.25 µM) on the expression level of HIF-1α on MCF-7 and LCC2 cells was examined via western blotting. In MCF-7 and LCC2 cells, 2-ME or TAM administration reduced the expression level of the HIF-1α protein in comparison to the control (Fig. 3). The inhibition of HIF-1α was more obvious with TAM/2-ME co-treatment than solitary treatment on both cells.

### Combining treatment using TAM and 2-ME elevates the level of apoptosis-related markers in MCF-7 and LCC2 cells

**Fig. 4:**
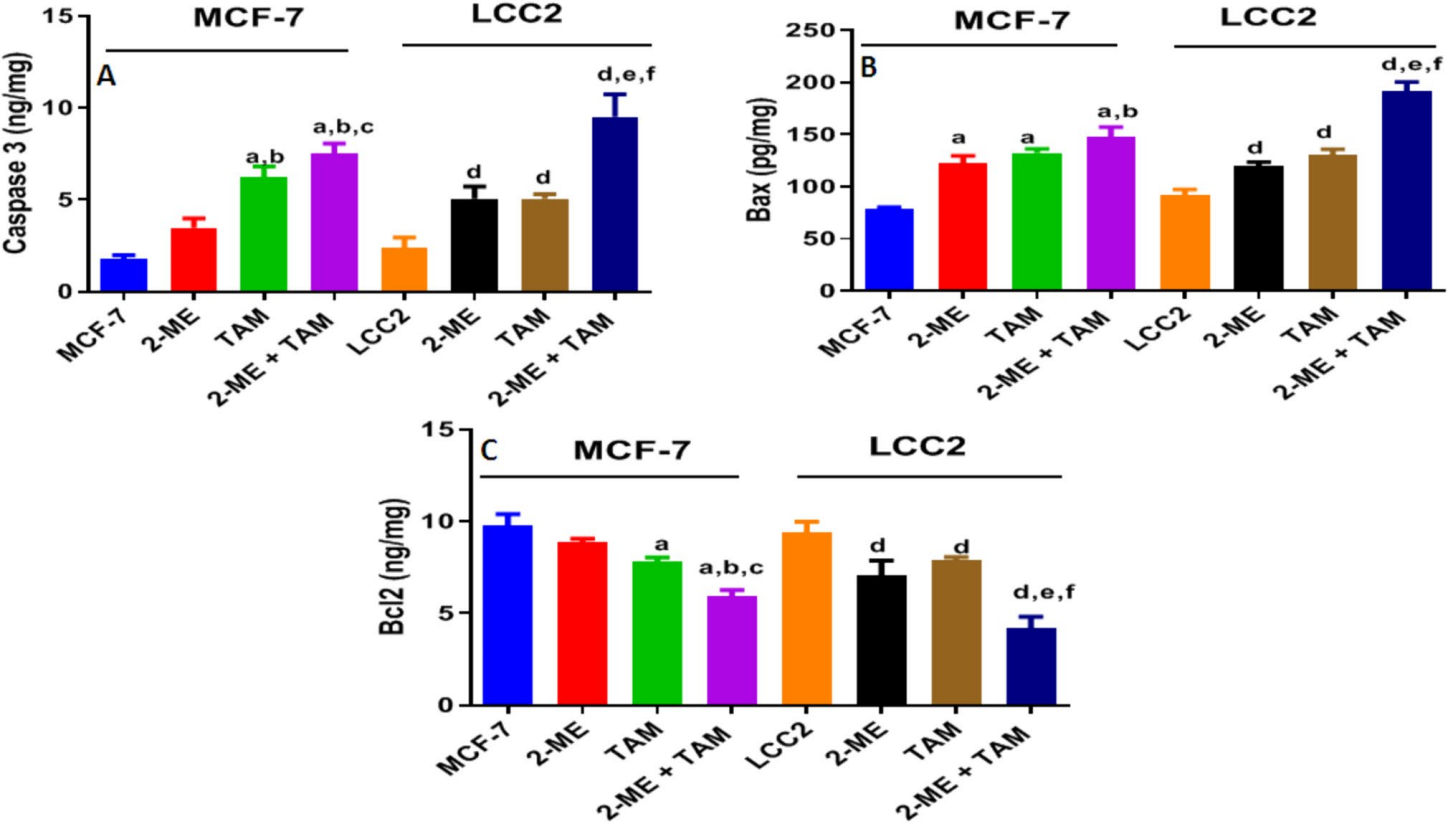
Co-treatment with TAM and 2-ME increases the level of apoptosis-related markers in MCF-7 and LCC2 cells. In MCF-7 and LCC2 CELLS, 2-ME/TAM concurrent treatment led to a rise in caspase 3, Bax, and a reduction in Bcl2. Two different concentrations of 2-ME and TAM were applied to MCF-7 and LCC2 cells, respectively, during a 48-h period. By using an ELISA, the levels of caspase 3 **A**, Bax **B**, and Bcl2 **C** in each cell were determined. The findings were presented as mean ± SD derived from 3 separate studies. “a” Significantly distinct from MCF-7 control, “b” Significantly distinguished from MCF-7 treated with 2-ME. At *P* 0.05, the following comparisons were found to be significant: “c” Notably different from MCF-7 treated with TAM, “d” Remarkably different from LCC2 control cells, “e” Significantly distinctive from LCC2 treated with 2-ME, and “f” Statistically different from LCC2 treated with TAM

In comparison to control or single treatment, concurrent treatment of MCF-7 and LCC2 cells with TAM (2.5 μM, 35 μM) and/or 2-ME (2.5 μM, 1.25 μM) results in a considerable rise in caspase-3 level (Fig. 4A) and Bax level (Fig. 4B), as well as a decrease in Bcl2 level (Fig. 4C). In-depth: In LCC2 cells, concurrent treatment with TAM and 2-ME elevated caspase-3 by 403%, whilst in MCF-7 cells, it elevated caspase-3 by 418%. Similarly, LCC2 cells showed a 207% rise in Bax following the addition of TAM and 2-ME, whereas MCF-7 cells showed an 189% increase. On the contrary, 44.5% less Bcl2 was detected in LCC2 cells after TAM and 2-ME were combined; Nevertheless, was 60% in MCF-7 cells. This finding also suggests that 2-ME and TAM co-treatment have a stronger impact on LCC2 cells than MCF-7.

### TAM/2-ME combined treatment lowered cholesterol and TG levels (mg/100 mg protein) in MCF-7 and LCC2 cells.

**Fig. 5:**
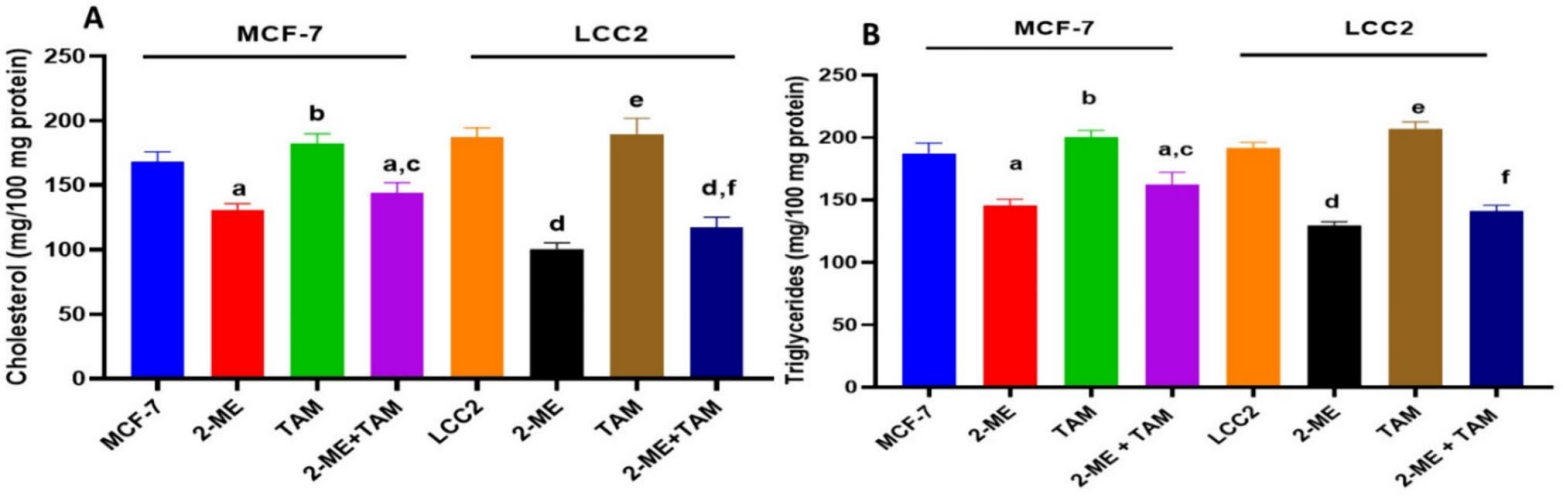
TAM/2-ME combination therapy reduced the amount of cholesterol and triglycerides (TG) per 100 mg of protein in MCF-7 and LCC2 cells. Following a combination with TAM and 2-ME, MCF-7 and LCC2 cells showed increased TG and cholesterol content (mg/100 mg protein). MCF-7 and LCC2 cells were exposed to 2-ME (5 μM and 2.5 μM, respectively) and TAM (5 μM and 17.5 μM, respectively) for 48 h. TG (**B**) and cholesterol (**A**) concentrations in MCF-7 and LCC2 cells that had undergone different treatments were measured using spectroscopic measurement The results of three separate studies (*n* = three) are computed as mean ± SD. Employing one-way ANOVA and Tukey’s post hoc multiple comparison tests, the statistical significance was evaluated. Statistically different from MCF-7 controls in “a” and “b” Statistically different from MCF-7 treated with 2-ME, “d” Significantly different from LCC2 control cells, “e” Significantly different from LCC2 treated with 2-ME and “f” Significantly different from LCC2 treated with TAM, at *P* < 0.05

As seen in Fig. 5A a combination of 2-ME and TAM considerably decreased cholesterol levels relative to the control and/or single-treated groups. The LCC2 cells exhibit a much lower proportion of decreased cholesterol content in TAM + 2-ME (62%), compared to MCF-7 cells (85%).

Like what was said previously, TG was considerably reduced in MCF-7 and LCC2 cells treated with TAM and 2-ME combination compared to control groups and/or single treatments (Fig. 5B). Furthermore, our results imply that LCC2 cells are more significantly affected by 2-ME/TAM co-treatment than MCF-7 cells.

## Discussion

Endocrine therapy is crucial in the treatment of BC, which is the most frequent malignancy in women, especially ER-positive BC accounts for more than two-thirds of all cases. Tamoxifen, an ER antagonist, is the most frequently used endocrine therapy to treat BC that are ER-positive. However, its efficacy is limited by the development of drug resistance [6]. Several factors were involved in TAM efficiency and the development of TAM resistance like metabolic reprogramming, oncogenic pathways, and receptor down-regulation [6, 25, 26]. Also, the hypoxia and its related factors including HIF-1α contributed to BC progression and TAM resistance. Due to inadequate perfusion, breast malignancies, and most tumors are hypoxic [27, 28]. The hypoxia-inducible transcription factor alpha-subunits, HIF-1 and HIF-2, accumulate and become active in response to hypoxia. The alpha-subunit forms a dimer with HIF-β to produce a transcription factor complex that moves to the nucleus where it binds to hypoxia-responsive elements (HREs) in the genome. The hypoxic transcriptome comprises genes involved in angiogenesis, glycolysis, cell survival, and proliferation, which may hasten cancer development [27, 28]. The BC cells grown under hypoxic conditions were resistant to TAM [29]. Hence, in this study, we aimed to explore the effect of targeting hypoxia factor HIF-1α by 2-ME and show its impact on TAM resistance.

The 2-ME is an endogenous metabolite of 17-estradiol (E2) and interacts with microtubules and ERs, however, it has a low affinity for ERs [13]. Additionally, 2-ME acts as an anti-angiogenic, anti-proliferative, and pro-apoptotic agent via decreasing HIF-1 protein levels and its transcriptional activity [30]. Its impact interferes with regular microtubule stability and function, and it is correlated with a decline in tubulin polymerization [31, 32].

In this study, both TAM and 2-ME in a single form decreased the viability of both resistant and sensitive BC cells. However, TAM IC_50_ is higher in resistant cells than in sensitive cells. This data indicated that TAM is effective in treating BC, but the development of resistance limited its application [6].

Further, the combination of 2-ME and TAM indicated a synergistic interaction on TAM-resistant cells at almost of tested concentrations and decreased the cell viability than a single treatment. The effect is more pronounced on TAM-resistant cells than on sensitive cells. For illustration, the CI for TAM/2-ME co-treatment ranges from 0.2 to 1.01 on TAM-resistant cells, while ranges from 0.62 to 1.21 on sensitive cells. This data collectively indicated an enhancement in the sensitivity and sensitization of TAM-resistant cells after co-treatment with 2-ME. This data were illustrated for the first time in this work. Besides, this result agreed with the previous research which indicated that the combination of 2-ME2 and gemcitabine caused a growth inhibition with major toxicity observed in the combination group in vivo and in vitro pancreatic cancer cells [33]. Also, another study explored that co-administration of paclitaxel and 2-ME reduces drug resistance [34].

Hypoxia is one of the many reasons for drug resistance, which involves several pathways. A crucial transcription factor known as HIF-1α controls how cells react to hypoxia. HIF-1α can cause angiogenesis, anaerobic glycolysis of tumor cells, tumor cell proliferation, invasion, and migration, as well as multidrug resistance [35]. In addition, BC patients with HIF-1α overexpressed are more likely to develop medication resistance, have a poor prognosis, and develop metastasis [36]. Thus, targeting HIF-1α is expected to overcome therapeutic resistance [35]. Accordingly, in our study, the protein level of HIF-1α was examined by western blotting. The data indicated that the TAM/2-ME co-treatment decreased the protein level of HIF-1α than solitary treatment with TAM or 2-ME. Thus, the enhancement and sensitization of TAM-resistant cells was mediated at least in part through inhibition of HIF-1α by 2-ME. Therefore, hypoxia plays an important role in the response of BC cancer cells to treatment with TAM and targeting of its key transcription factor alleviates the drug resistance. Furthermore, the finding suggests that the regulation of a crucial gene involved in lipid metabolism is at least partially responsible for how TAM affects BC. Since HIF-1α must be suppressed by 2-ME for LCC2 cells to be responsive to TAM.

The HIF-1α controls various processes of cancer cells including cell growth, proliferation, invasion, apoptosis, and autophagy. Thus, targeting of HIF-1α alters these processes and is accompanied by cell death [37]. Subsequently, in this study, the level of apoptotic (caspase 3), pro-apoptotic (Bax), and anti-apoptotic (Bcl2) marker levels was measured by ELISA. The results demonstrated that the treatment with TAM or 2-ME increased the level of caspase 3 and Bax while decreasing the level of Bcl2 an effect that was more pronounced after co-treatment with TAM/2-ME than solitary treatment. Also, the degree of increase in caspase 3 and Bax or the decrease in Bcl2 was more obvious in TAM-resistant cells than in sensitive cells. This result was in accordance with the previous research which indicated that inhibition of HIF-1α gene expression by ceranib-2 induces apoptosis in HepG2 cells [38]. Additionally, the study of *Tang *et al. discovered that 2-ME therapy-induced apoptosis and inhibited osteosarcoma cells, which was accompanied by lower levels of Bcl-2 expression and elevated caspase-3 expression [39]. Furthermore, *Alhakamy *et al. [40] indicated that treatment of prostate cancer cells with 2-ME increased the rate of apoptosis accompanied by an increased level of BAX and decreased the level of Bcl2 [40].

HIF1 is a master transcription factor and regulates the expression of many genes involving many aspects of biology. In addition to HIF1’s roles in glucose metabolism and angiogenesis, numerous studies have revealed an emerging role of HIF1 in controlling lipid homeostasis such as lipid uptake, synthesis……etc.. and due to the emerging role of lipids in cancer cells to perform cell membrane and signaling the authors measure the content of cholesterol and TG [41, 42]. Our results indicated that treatment with 2-ME alone or in combination with TAM decreased the lipid content of both TAM-sensitive and resistant cells, the effect was more pronounced in TAM/2-ME co-treatment than solitary treatment. Also, the effect was more pronounced on TAM-resistant cells than on sensitive cells. In contrast, the TAM solitary treatment increases the lipid content of both cells. This data illustrated the involvement of lipids in the development of TAM resistance and the reduction of lipid content and targeting lipid metabolic reprogramming of cancer cells is one of the mechanisms of sensitization of TAM-resistant cells after co-treatment with 2-ME. This data agree with *Ibata *et al. [43] who showed that treatment with 2-ME reduced the lipid content of HepG2 cells and explained that via upregulation of ATP-binding cassette transporter A1 (ABCA1) and increasing lipid efflux through PI3K/Akt/FoxO1 pathway [43].

Collectively, however, TAM co-treatment with 2-ME sensitized TAM-resistant cells, increased apoptotic markers, decreased lipid content, and decreased HIF-1α, this data give a basic concept for further in vivo investigation before clinical trial.

## Conclusion

Our findings show that 2-ME increased TAM cytotoxicity and overcame TAM resistance via suppression of HIF-1α, along with an increase in the apoptosis marker caspase-3, an increase in pro-apoptotic Bax, and a decrease in Bcl2 levels. Additionally, a decrease in TG and cholesterol content was observed. Additionally, 2-ME reduces the effective dose of TAM while sensitizing resistant cells to TAM. Accordingly, TAM/2-ME co-treatment may be a potential therapeutic strategy to overcome TAM resistance and improve the therapeutic outcomes for BC patients.

### Supplementary Information

Below is the link to the electronic supplementary material.

Supplementary file1 (DOCX 17 KB)

## Data Availability

No datasets were generated or analysed during the current study.
