# Supplementary material for: 2-methoxyestradiol sensitizes tamoxifen-resistant MCF-7 breast cancer cells via downregulating HIF-1α
Source: Med Oncol. 2024 Aug 21;41(9):232. doi: 10.1007/s12032-024-02471-w (PMC11339102; doi:10.1007/s12032-024-02471-w)
Supplement: Supplementary file 1 — Supplementary file1 (DOCX 17 KB) [file 12032_2024_2471_MOESM1_ESM.docx]

**Supplementary Table 1: 2-ME improves TAM cytotoxic effect on MCF-7 and LCC2 cells.**

| **MCF-7** | | | **LCC2** | | |
| --- | --- | --- | --- | --- | --- |
| **2-ME (µM)** | **TAM (µM)** | **Viability % from control mean** ± SD | **2-ME (µM)** | **TAM (µM)** | **Viability % from control mean** ± SD |
| 1.25 | 2.5 | 66.96 ± 5.6 )p<0.001) | 1.25 | 35 | 18.7 ± 4.3 )p<0.05) |
| 2.5 | 2.5 | 49.7 ± 4.5 )p<0.001) | 2.5 | 35 | 15.6 ± 5.7 )p<0.05) |
| 5 | 2.5 | 45.10 ± 4.6 )p<0.001) | 5 | 35 | 12.13 ± 4.2 )p<0.05) |
| 7.5 | 2.5 | 39.08 ± 2.2 )p<0.001) | 7.5 | 35 | 11.2 ± 3.0 )p<0.05) |
| 10 | 2.5 | 32.8 ± 3.6 )p<0.001) | 10 | 35 | 6.7 ± 1.7 )p<0.01) |
| 1.25 | 5 | 60.98 ± 16 )p<0.05) | 1.25 | 17.5 | 45.6 ± 16.5 )p<0.05) |
| 2.5 | 5 | 48.03 ± 11.3 )p<0.01) | 2.5 | 17.5 | 34.8 ± 4.4 )p<0.001) |
| 5 | 5 | 41.9 ± 9.7 (p<0.05) | 5 | 17.5 | 30.8 ± 8.3 )p<0.05) |
| 7.5 | 5 | 30.8.4 ± 10.5 (p<0.05) | 7.5 | 17.5 | 27.9 ± 7.4 )p<0.05) |
| 10 | 5 | 21.2 ± 7.9 (p<0.05) | 10 | 17.5 | 26.2 ± 5.8 )p<0.05) |
